# Dye Adsorbent from Natural Rubber Latex Foam: Efficiency and Post-Utilization

**DOI:** 10.3390/polym17010106

**Published:** 2025-01-02

**Authors:** Abdulhakim Masa, Nureeyah Jehsoh, Nabil Hayeemasae

**Affiliations:** 1 Rubber Engineering Program, Department of Interdisciplinary Engineering, Faculty of Engineering, Prince of Songkla University, Songkhla 90110, Thailand; abdulhakim.m@psu.ac.th; 2 Department of Rubber Technology and Polymer Science, Faculty of Science and Technology, Prince of Songkla University, Pattani Campus, Pattani 94000, Thailand; nureeyah.psu56@gmail.com

**Keywords:** natural rubber latex foam, Methylene Blue, Alizarin Yellow, dye removal, foam, antibacterial foam

## Abstract

This study examined the feasibility of using natural rubber (NR) latex foam as a dye adsorbent and antibacterial foam. The dyes used in this experiment were Methylene Blue (MB) and Alizarin Yellow (AY). Foams with that optimum density were further evaluated for adsorption isotherm, kinetics, and thermodynamic data. The dye adsorption occurred in two steps: the initial and the stabilized stages where an increase in dye concentrations boosted the adsorption capacity. Based on the prediction, the maximum adsorptions of MB and AY from the solution were 3.15 and 10.31 mg/g, respectively. The Langmuir isotherm fits better with the adsorption of MB while AY is better matched by the Freundlich isotherm. Moreover, the adsorption behavior fits well with the pseudo-second-order model. MB took much longer to reach the stabilized stage, especially at high dye concentrations. The thermodynamic study revealed that physical adsorption accounted for most of the adsorption. Later, the foam after use as an adsorbent was further utilized as an antibacterial foam. Based on the qualitative and quantitative aspects, the results indicate that the dye-carrying foam could inhibit the growth of both Gram-positive and Gram-negative bacteria. It can be concluded that NR latex foam can be applied as a dye adsorbent and further utilized as an antibacterial foam.

## 1. Introduction

Since the earliest days, people have made great efforts to use natural resources to create beautiful and interesting items. They have attempted to use a variety of natural resources to produce dyes. Textile industries have colored objects with a variety of dyes to give them an appealing appearance. The textile industry uses a lot of chemicals and water for a lot of different reasons. Although the textile industry is known to strengthen a country’s economy, progress, and prosperity, it also emits dyes into water systems, which harms the environment [1,2,3]. The resulting wastewater, or effluent, is mostly contaminated by colorants that damage aquatic plants and animals and cause other environmental issues. Dyes are major pollutants found in the effluents from industrial sectors, including food, paper, textile, plastic, and cosmetics [4]. Dyes prevent light and oxygen from diffusing into the water by creating a foam on the water surface, as a result of their surfactant characteristics. This interferes with photosynthesis and respiration, impacting the biological processes of aquatic flora and fauna negatively [5,6]. In addition, most of the dyes are poisonous or strongly oncogenic [7,8], and contaminate the water even at minute concentrations.

Many adsorbents have been explored to remove dyes from water, including chitosan [9], activated carbon [10,11], surfactant-modified sepiolite [12], bentonite [13], silica [14], and clay [15], among others. Compared to other chemical and physical approaches, adsorption is thought to be the most efficient technique for removing colorants from wastewater [16]. Natural polymers such as chitin, chitosan, starch, and cellulose are becoming more and more popular as adsorbents because they are abundant, non-toxic, environmentally friendly, and highly efficient [17,18,19]. However, due to high operating costs, reusing, and detachment problems after various uses are still limited [20,21]. Moreover, bio-adsorbents would relatively sink when used in water. This is simply responsible for their specific gravity. Then, scientists are developing new materials that can effectively anchor dye moieties from wastewater.

Natural rubber foam (NRF) is an interesting rubber product that consists of rubber and gas phases. Because of it is lightweight, floats on water, is an excellent thermal insulator, and is able to absorb sounds, NRF has found widespread use [22,23]. There are various ways to generate NRF. The natural rubber (NR) latex technique, applying NR in liquid form, is typically used to make NRF [24]. As the rubber foam is porous, it has excellent adsorption properties. As such, this study examined using natural rubber foam to adsorb dyes. Due to the cellular structure of NR, dyes might adhere through physical adsorption in the pores of the NRF. However, one problem encountered when using dye adsorbents is the eventual removal of the adsorbent, as most adsorbents have a high specific gravity post-adsorption and tend to settle, making recovery difficult. Although dye adsorption is physical, it is reversible, and the adsorbed dye material remains. The dye-adsorbing material may be returned to the environment after use. Therefore, studies have sought to demonstrate materials capable of adsorbing dyes and of reuse after that. Rubber foam appears advantageous for use as an adsorbent. Storage is easy due to the low specific gravity, and it also reduces the sedimentation tendency.

In addition, this study also presents the idea of reutilizing adsorbent foams in antibacterial applications. Dyes can affect aquatic organisms. The charged colorants can destroy the protein layer in bacteria, destroying the cell wall and preventing the cells from dividing. The authors considered applying dye-adsorbent foams as antibacterial sponges to be an interesting solution for making further use of them. This approach can be utilized on developing antibacterial foams, having the advantage of a low cost.

## 2. Experimental Design

### 2.1. Materials

The 60% dry rubber content High Ammonia centrifuged latex (60% HA) was acquired from Yala Latex Industry, Co., Ltd., Yala, Thailand. The measured quality indicators of this HA latex were total solids content (61.4%), dry rubber content (60%), alkalinity (0.54%), mechanical stability time (875 s), and volatile fatty acids (0.06). Methylene Blue (MB) and Alizarin Yellow (AY) were the representative dyes in this study. MB is classified as a cationic dye while AY is an anionic dye. MB and AY were supplied by POCH S.A., Gliwice, Poland. The buffer solution at pH equal to 4.01 ± 0.01, 7.00 ± 0.01, and 10.01 ± 0.01 were used for control solution. These were supplied by Thermo Fisher Scientific, Inc., Singapore. In addition, 50% Zinc dithiocarbamate (ZDEC), 50% zinc 2-mercaptobenzothiazole (ZMBT), 50% WingstayTM L, 50% sulphur, 15% diphenyl guanidine, and 50% sodium silicofluoride were supplied by Siamnavakam Co. Ltd., Nonthaburi, Thailand.

### 2.2. Preparation of the Natural Rubber Latex Foam

The lab-scale formulation for the natural rubber latex foam is displayed in Table 1. The components in dispersion or emulsion forms were incorporated alongside the HA latex. The Dunlop process was used to prepare the natural rubber latex foam. First, a cake beater was filled with a specific amount of natural rubber latex (HA-type), and ammonia was gradually evaporated for 3 min. Subsequently, 20% potassium oleate, 50% ZDEC, 50% ZMBT, 50% Wingstay™ L, and 50% sulfur were added. The beating speed was increased and maintained until the desired foam volume was achieved (5 min). From this step onwards, the foaming volumes varied depending on the experimental design. In the first series, the foaming percentages varied from 20–80% of the original volume of beater (see Table 2), while a foaming volume of 60% was selected for the second series. The foaming percentage can be calculated from Equation (1).

Foaming percentage = (Volume of Foam (mL))/(Volume of Beater (mL)) × 100(1)

Next, 50% DPG and 50% ZnO were added, followed by a quick addition of the gelling agent (20% SSF), and the mixture was beaten for another 1 min. Finally, the un-gelled foam was immediately poured into the aluminium mould and allowed to be set for 2 min at ambient temperature. The gelled foam was cured for 2 h at 100 °C in a hot air oven. To remove soap and unreacted substances, the dried foam was thoroughly cleaned with water after being removed from the mould. The cured natural rubber latex foam was washed and dried for 4 h in a hot air oven at 80 °C.

### 2.3. The Adsorption Capacities (q_e_) and Corresponding Adsorption Kinetics of Natural Rubber Latex Foams

**Figure 1:**
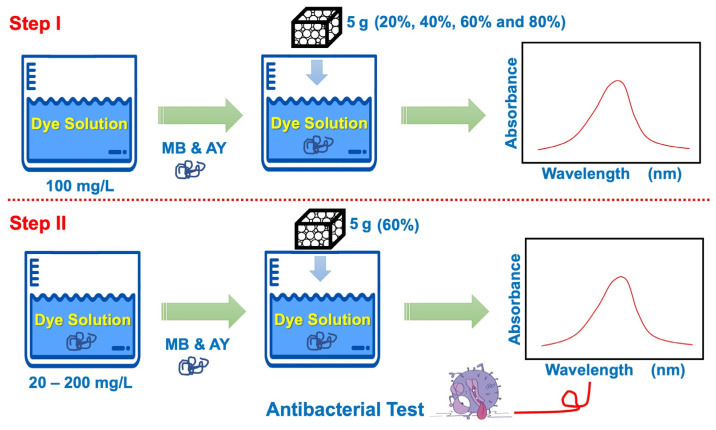
An illustration of examining the effects of the foaming volume (Step I) and initial concentration (Step II) on MB and AY removal.

Batch adsorption was used to examine the adsorption capacity (*q_e_*) for MB and AY by the natural rubber latex foam. The calculation of *q_e_* was based on the report of Cooney [25]. Ultraviolet–visible (UV–Vis) spectrophotometry at approximately 660 nm (MB) and 360 nm (AY) wavelengths was used to determine the amounts of residual MB and AY. For statistical analysis, each experimental run was replicated three times, and the mean values are reported. The calculated adsorption capacity (*q_e_*) was based on the calibration curve of absorbance as a function of the initial concentration of dye. The MB and AY solutions had initially 5 g per 100 mL weight per volume concentration. After adding 5 g of natural rubber latex foam to 100 mL of MB or AY solution, the mixture was shaken constantly. Then, 1 mL samples were taken at pre-arranged intervals using a syringe. Ultraviolet–visible (UV–Vis) spectrophotometry was applied to these. The experiments fell into 2 sets (see Figure 1). Step I assessed the effects of foaming volume (20%, 40%, 60%, and 80%) on *q_e_*. A 100 mg/L concentration of both MB and AY was chosen to determine the optimum natural rubber latex foaming volume or foam density. The natural rubber latex foam with the optimum density or foaming volume (60%) was chosen for the next series based on its *q_e_* (Step II). Then, initial concentrations of 20 to 200 mg/L were applied. Both MB and AY were dissolved in deionized water. The *q_e_* at various contact times was determined up to 24 h. All experiments were repeated thrice to ensure reproducibility, and the averages are reported. The *q_e_* was calculated using Equation (2):

(2) $$q_{e}=\frac{V\left(C_{0}-C_{e}\right)}{W}$$

where *q_e_* is the adsorption capacity (mg/g), *V* is the volume of the solution (100 mL), *W* is the mass of the adsorbent (approximately 5 g), and *C*_0_ and *C_e_* are the initial and equilibrium concentrations of the MB or AY (mg/L). *C*_0_ and *C_e_* were calculated using a linear regression fit of UV absorbance and initial concentration.

Kinetics of adsorption and adsorption isotherms were assessed using the data. The MB and AY solutions were subjected to kinetic tests at initial concentrations ranging from 20 to 200 mg/L. Pseudo-first-order and pseudo-second-order kinetic models were trialed for MB and AY solutions [25]. Equation (3) gives the pseudo-first-order model using the Lagergren equation:

(3) $$\frac{dq_{t}}{d_{t}}=k_{1}\left(q_{e}-q_{t}\right)$$

This can be re-written into Equation (4):

(4) $$ln\left(q_{e}-q_{t}\right)={lnq}_{e}-k_{1}t$$

The pseudo-second-order model according to Ho and Mc Kay [26] is shown in Equation (5):

(5) $$\frac{dq_{t}}{d_{t}}=k_{2}{\left(q_{e}-q_{t}\right)}^{2}$$

This can be re-written into Equation (6):

(6) $$\frac{1}{q_{t}}=\frac{1}{k_{2}q_{e}^{2}}+\frac{1}{q_{e}}$$

Equation (7) shows Weber and Morris’ [27] intraparticle diffusion model:

(7) qt=kpt12+C

where *q_t_* is the amount of MB or AY adsorbed (mg/g) at any time (*t*), *k*_1_ is the rate constant of the pseudo-first-order (min^−1^), *k*_2_ is the rate constant of the pseudo-second-order (g/mg·min), *k_p_* is a rate constant of intraparticle diffusion (mg/g·min), and *C* is a constant in any experiment (mg/g).

The Langmuir and Freundlich isotherms are alternative models that were tested for fit with the adsorption data, to determine the adsorption mechanism. The Langmuir adsorption isotherm is predicated on the idea that there are no interactions between adsorbed molecules and that the adsorbent’s surface has a uniform distribution of a finite number of active sites. The Langmuir equation can be expressed as follows:

(8) $$q_{e}=\frac{q_{0}K_{L}C_{e}}{1+KC_{e}}$$

This can be re-written into Equation (9):

(9) $$\frac{1}{q_{e}}=\frac{1}{q_{m}}+\frac{1}{K_{L}q_{m}C_{e}}$$

where *q_m_* and *K_L_* are the Langmuir constants, representing the maximum of *q_e_* (mg/g) and the constant of adsorption equilibrium (L/mg).

For non-ideal adsorption, which happens in systems with heterogeneous surface energy, the Freundlich isotherm is a candidate model. It assumes that adsorption takes place at sites with varying energies. The Freundlich equation is as follows:

(10) qe=KFCe12

This can be re-written into Equation (11):

(11) $$logq_{e}=logK_{F}+\frac{1}{n}logC_{e}$$

where *K_F_* and *n* are Freundlich constants of *q_e_* and adsorption intensity.

### 2.4. Antibacterial Performance of the Natural Rubber Latex Foam

Both qualitative and quantitative techniques were used to evaluate the antibacterial performance. The qualitative method followed ASTM E2149 [28], while ASTM E2315 [29] was referred to as the quantitative method. As for the first experiment, the natural rubber latex foam was sandwiched between MHA layers and deposited in different locations on Muller Hinton Agar (MHA) plates under an aseptic environment, both with and without adsorbed dye. Sterilized Petri dishes were filled with 15 mL of MHA in an aseptic manner to generate the lowest layer. The foam’s surface was coated with 10 mL of MHA to form the thin top layer. The optical density (OD) at 600 nm was used to calculate the bacterial concentration. The OD value 0.3 corresponded to 1 × 10^8^ CFU/mL bacterial concentration. Amounts of 100 µL of *Escherichia coli* (*E. coli*) bacterial solution and of *Staphylococcus aureus* (*S. aureus*) were inoculated onto MHA plates and evenly spread. The inoculated agar plates were incubated at 37 °C for 24 h for inhibition zone measurement.

For quantitative analysis, the test culture was grown on nutrient agar (NA) medium and incubated at 37 °C for 18 to 24 h. Separate colonies were then placed in Muller Hilton Broth (MHB) and incubated for 3 h at 37 ± 0.5 °C. The McFarland criterion of 0.5 was used to compare the turbidity. Then, 100 µL of prepared bacteria was added to 9 mL of MHB medium containing the natural rubber latex foam test samples with and without adsorbed dye. The sample was incubated at 37 ± 0.5 °C for 0 and 24 h. Then, the bacteria were diluted 10-fold with 0.85% normal saline, spread onto NA medium, and incubated overnight at 37 ± 0.5 °C. The number of colonies of bacteria that survived (CFU/mL) was recorded. To enable comparison, the outcomes of control experiments were also noted. Additionally, the reduction percentages are given.

## 3. Results and Discussion

### 3.1. Foaming Volume, Density, and Corresponding Adsorption Capacity of Natural Rubber Latex Foam

**Figure 2:**
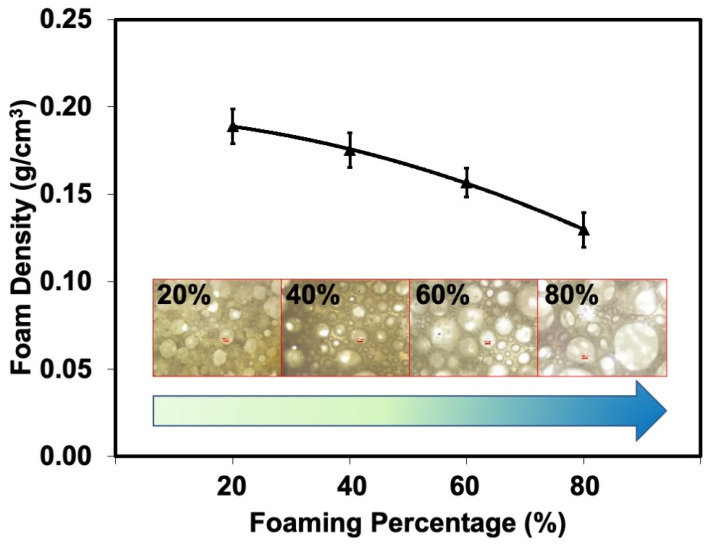
The foam densities and corresponding optical images of the natural rubber latex foams prepared to various foaming volumes.

The dye adsorbency of natural rubber latex foam depends on two types of factors: physical and chemical characteristics. Here, the focus was on the physical characteristics of natural rubber latex foam. The experimental design manipulated the density of the natural rubber latex foam. An increase in the air phase increased the number of cells and the porosity of the foam. Therefore, the foam density can be controlled by fixing the foaming volume while beating the foam. The effect of foaming volume on the foam density of natural rubber latex foam is shown in Figure 2. As expected, an increase in foaming volume reduced the foam density as the fraction of air phase increased. The porosity of the foam increased accordingly. Therefore, the foam weight was reduced for a given volume. This corresponds well with the optical images shown in Figure 2. As seen, uniformly distributed cell sizes were obtained at low foaming volumes. However, the cell size increased as the foaming volume increased. Increasing the foaming volume increased the air phase. This increased the porosity of the foam. An increase in cell volume is expected to increase the contact region throughout the rubber phase, increasing the diffusivity of the dye. This is an important factor that impacts the dye removal efficiency.

Step I was to optimize the foaming volume for maximal *q_e_*. The 100 mg/L MB and AY were the representative dyes in this case. A higher foaming increased the *q_e_*, suggesting that it effectively increased the dye removal from MB and AY solutions. Table 3 clearly shows that the adsorbed amounts of MB and AY increased with foaming volume, so that they were the highest at 60% foaming volume. At a low foaming volume, the cell size and porosity remain well-distributed, giving a good diffusion of the dye solution through the foam sample. However, when the highest foaming volume was used (80%), the adsorbed amount no longer increased but remained about the same. This may be due to the heterogeneous distributions of the cell size and porosity. As the pores became bigger, the surface area was reduced, and the adsorption was then decreased. Xia et al. [30] suggested that the adsorption performance is linked to factors like heterogeneous adsorbent structures. These structures may have various surface areas, which can cause uneven dye distribution and decrease adsorption in certain cases. Therefore, only the natural rubber latex foam prepared to 60% foaming volume was studied further.

**Figure 3:**
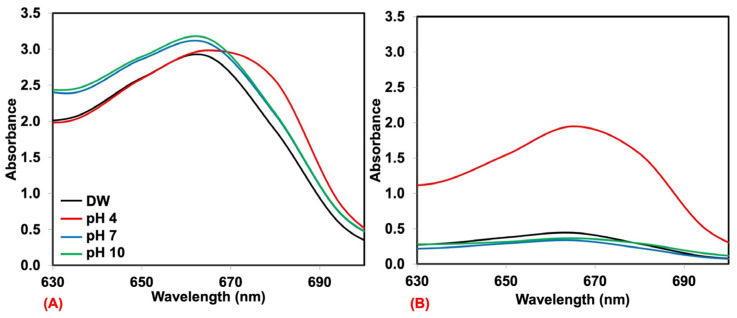
Comparison of the UV absorbance of MB at the concentration of 25 mg/L dissolved in various buffer solutions, namely, deionized water (DW), pH 4, pH 7, and pH 10 before (**A**) and after (**B**) adsorption by natural rubber foam.

**Figure 4:**
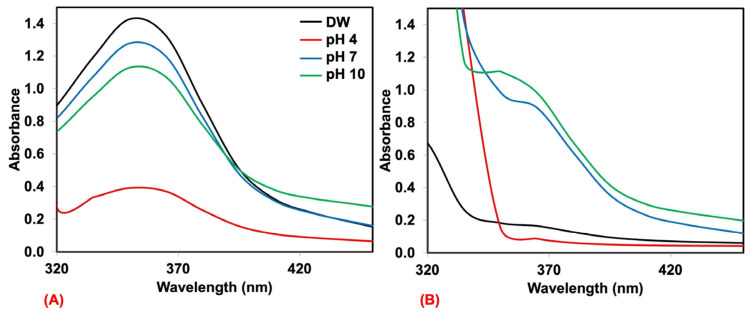
Comparison of the UV absorbance of AY at the concentration of 25 mg/L dissolved in various buffer solutions, namely, deionized water (DW), pH 4, pH 7, and pH 10 before (**A**) and after (**B**) adsorption by natural rubber foam.

In general, dyes are sensitive to the pH of the solution, and the dye adsorption at pH 4.01 ± 0.01, 7.00 ± 0.01, and 10.01 ± 0.01 was also observed. Here, the natural rubber foam prepared at 60% foaming volume was used for this case. The UV-absorbance of MB and AY solutions before and after being adsorbed are shown in Figure 3 and Figure 4. Here, the as-received MB and AY solutions were used at 25 mg/L to obtain an acceptable absorbance range. It is seen that both dyes are sensitive to the pH ranges. The UV absorbance of MB is shown in Figure 3A,B. It is seen that the UV absorbance of MB is more or less the same before and after adsorption by natural rubber except for the MB in the pH 4 buffer solution. MB is a cationic dye, and the positive charge is stable in acidic environments [31]. Therefore, the color remained blue even after the adsorption by natural rubber foam. This would limit the use of natural rubber foam as MB removal under acidic conditions. As for AY, it is an anionic dye; the yellow color might be changed under acid conditions as it is more stable in basic conditions [32]. As can be seen from Figure 4A,B, the UV absorbance of AY in the pH 4 buffer solution is lower than other solutions. Upon adsoption by natural rubber foam, all samples showed a significant reduction in the UV absorbance, showing the good efficiency of natural rubber foam against AY. Moreover, dye removal under various pHs is something to ponder in the study of dye adsorbent materials.

### 3.2. Effect of MB or AY Concentration on Removal Efficiency by Natural Rubber Latex Foam

**Figure 5:**
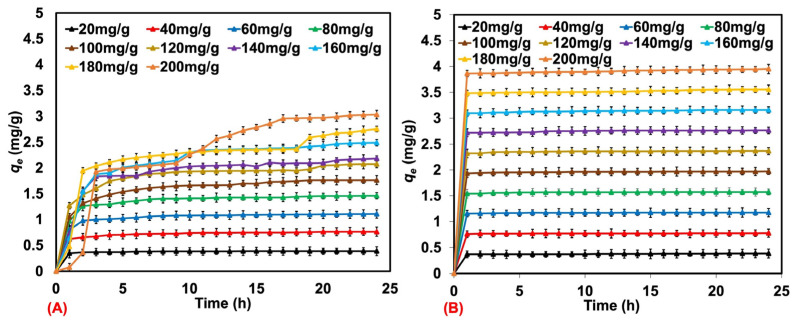
The amounts of MB (**A**) and AY (**B**) adsorbed by the natural rubber latex foam from various initial concentrations.

**Figure 6:**
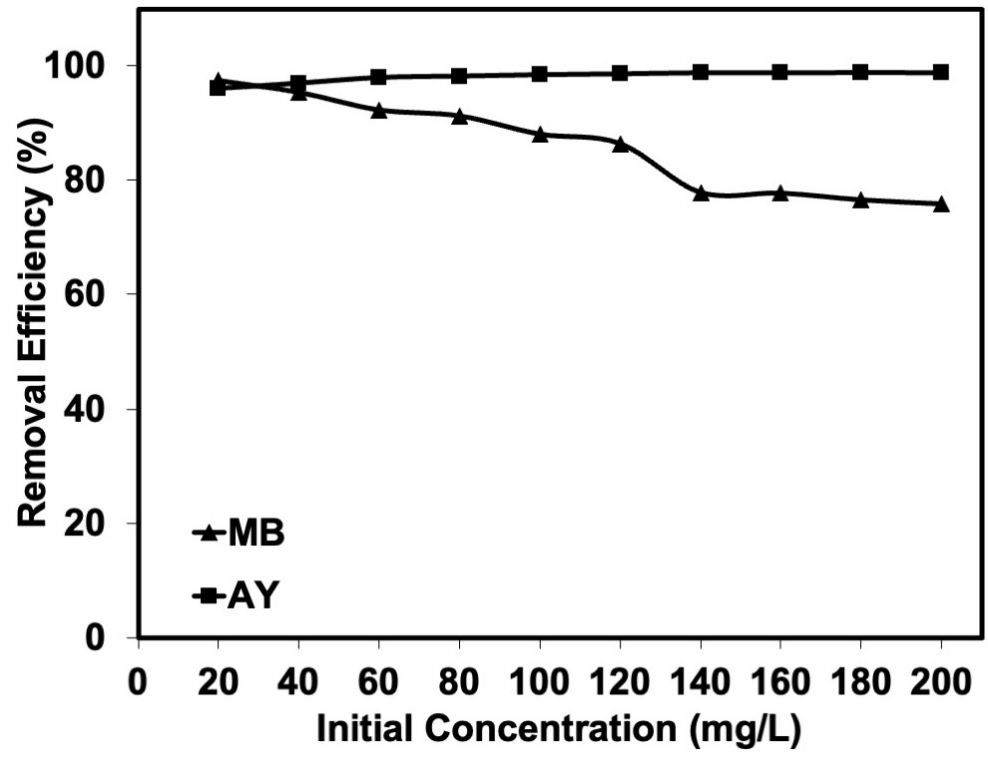
Removal efficiencies from MB and AY solutions by natural rubber latex foam.

As mentioned previously, the natural rubber latex foam prepared at 60% foaming volume was selected for step II. The effects of initial MB and AY concentrations among 20, 40, 60, 80, 100, 120, 140, 160, 180, and 200 mg/L on the removal efficiency by natural rubber latex foam were determined. Figure 5A,B show the amounts of MB and AY adsorbed with the various initial concentrations. Natural rubber latex foam exhibited good adsorption in both MB and AY solutions. As for the AY solution, the *q_e_* could reach a maximum with a contact time shorter than 2 h. This is in contrast to the MB solution for which the maximum *q_e_* was reached at different contact times. The MB adsorption took a much longer time when a high initial concentration was tested. The natural rubber latex foams could adsorb more AY than MB. The removal efficiency of AY remained constant across the initial concentrations (see Figure 6), while MB adsorption tended to decrease especially at a high concentration of MB.

The different dye adsorption efficiencies may be due to different diffusion rates of the dyes. Moreover, their solubilities in water differ. The water solubility of a dye, expressed in grams per liter (g/L), indicates the maximum amount of dye that can be dissolved in one liter of water at a specific temperature and pressure. This provides insights into the dye’s ability to dissolve in water and form a homogeneous solution. The water solubility of MB is 40 g/mL, while that of AY is 12 g/mL. It can be seen that MB has better water solubility than AY. Natural rubber latex foam is an open-cell cellular structure, with the foam characterized by interconnected voids or cells, allowing air, liquids, or gases to pass through the material easily. The high water solubility of MB may compete with absorption by the adsorbent. Therefore, the water solubility of the selected dye is an important factor that determines the dye adsorption capacity. The experimental results found are consistent with the adsorption kinetics, which are discussed in the next section.

**Figure 7:**
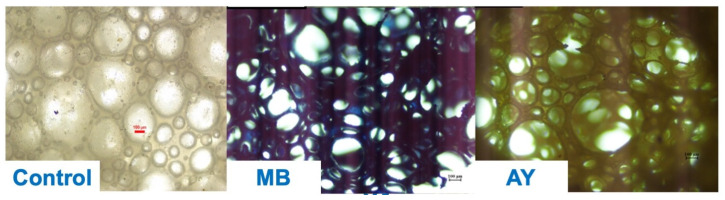
Optical images of the natural rubber latex foams before (control) and after MB and AY absorption.

Figure 7 comprises optical images of the natural rubber latex foam before and after adsorbing MB and AY. The white areas are the air phase, whereas the colored areas are the rubber phase. The MB and AY were adsorbed throughout the rubber. The porous foams had an increased removal efficiency due to the diffusion of MB and AY solutions throughout the sample.

### 3.3. The Kinetics of Absorption by Natural Rubber Latex Foam

**Figure 8:**
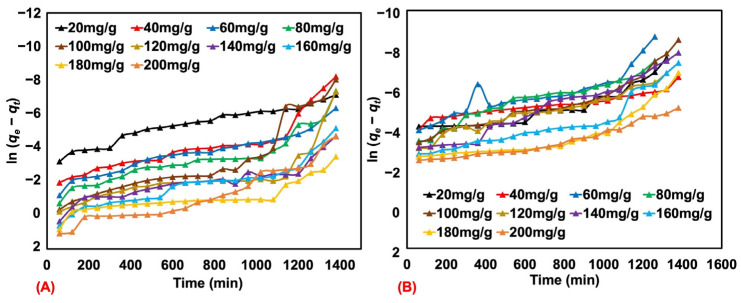
The pseudo-first-order plots for MB (**A**) and AY (**B**) adsorbed by natural rubber latex foam from various initial concentrations.

**Figure 9:**
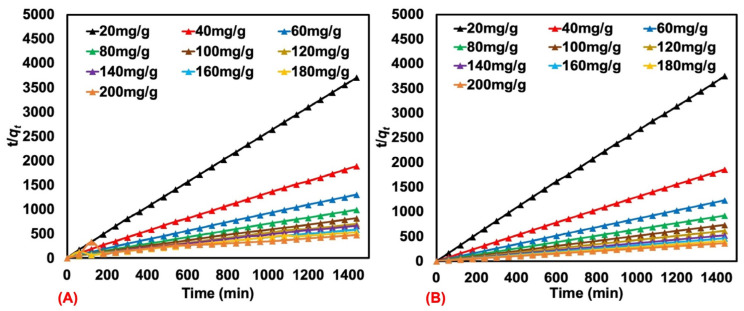
The pseudo-second-order plots for MB (**A**) and AY (**B**) adsorbed by natural rubber latex foam from various initial concentrations.

The kinetic behavior was further studied to identify the type of adsorption and other details. The pseudo-first-order model presumes that physisorption restricts the adsorption rate of the particles onto the adsorbent, while the pseudo-second-order model considers both physisorption and chemisorption as rate-limiting mechanisms of the process. The rate of adsorption is described by both models. In general, the adsorption by a porous material occurs in three steps, namely, (a) diffusion at the outer layer, (b) adsorption, and (c) diffusion within the pores. The pseudo-first-order (see Figure 8A,B) and pseudo-second-order (see Figure 9A,B) adsorption models were tested for natural rubber latex foam at various MB and AY concentrations. The quantity that linearizes the pseudo-first-order model does not appear linear, so this model is not appropriate. In contrast, linear behavior is exhibited in plots for pseudo-second-order adsorption. This is clear also from the coefficient of determination (R^2^) in Table 4 and Table 5, with higher values preferring the pseudo-second-order model over the pseudo-first-order one.

Here, the pseudo-second-order exhibits a good match with data, corroborating the uptake both physically and chemically. This is consistent with most studies using different sorbents. For example, Hayeeye et al. [33] used gelatine and activated carbon to adsorb Rhodamine B. They summarized that the adsorption was well-matched by the pseudo-second-order model and concluded that both physical and chemical adsorption occurred. The data from the two fitted equations were used to estimate the adsorption rate constants, where *k*_1_ indicates the rate constant for pseudo-first-order adsorption, while k_2_ is for the pseudo-second-order adsorption (see Table 4 and Table 5).

It is observed that the rate constants *k*_1_ and *k*_2_ showed different behaviors. k_1_ exhibited a more fluctuating trend, simply because the adsorption behavior was not steady in the linearized plot for the pseudo-first-order adsorption model. However, this does not happen for *k*_2_, which tended to decrease with MB and AY concentrations. As can be seen from the adsorption efficiency (see Figure 5), the absorbed amount *q_t_* was high at the early times (<2 h); this shows a faster rate of physical and chemical adsorption in the beginning. This may affect the MB and AY solutions faster for the low initial concentrations. This is verified later by assessing the rate constants at both the initial and stable stages of adsorption. Another possible reason may be the amount of adsorbent used. Since a fixed amount of sorbent was used at any concentration, the adsorption may be pre-saturated at a low initial concentration. The rate of adsorption, therefore, decreases or does not change much according to the initial concentration of the dye.

**Figure 10:**
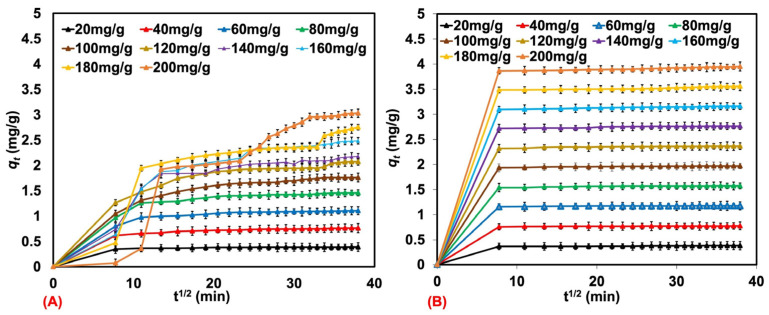
The amount qt of MB (**A**) and AY (**B**) that natural rubber latex foam has adsorbed versus *t*^1/2^.

The intraparticle diffusion model was also evaluated to study the rate constant at each adsorption step (see Figure 10A,B). The adsorbed amount at any time (*q_t_*) was plotted as a function of the square root of time (*t*^1/2^). Here, two adsorption steps can be observed, namely, the initial and stabilized stages of adsorption. The rate of each step can be interpreted by *ki*_1_ and *ki*_2_, shown in Table 6. *ki*_1_ indicates the intraparticle diffusion rate constant for the initial adsorption stage, while *ki*_2_ is for the rate constant at the stabilized stage. *ki*_1_ was higher than *ki*_2_, which is due to the instantaneous initial adsorption. The second step is the adsorption equilibrium caused by the decreasing dye concentration in the solution. It results from diffusion between particles and within the pores. In addition to this, the *ki*_2_ of MB was relatively higher than the *ki*_2_ of AY. This means that the adsorption of MB in step II was still active and not saturated like in the case of AY. As mentioned earlier, MB has better water solubility than AY, and the water solubility is responsible for this phenomenon.

### 3.4. The Adsorption Isotherms for Natural Rubber Latex Foam Adsorbent

**Figure 11:**
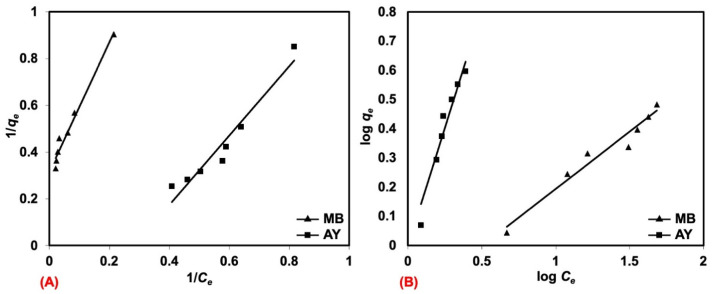
The linearizing plots for (**A**) Langmuir and (**B**) Freundlich isotherms, when natural rubber latex foam is adsorbing MB and AY.

Two kinds of isotherm equations, namely, the Langmuir and Freundlich isotherms, were tested to match the adsorption data and to indicate the mechanisms of adsorption. The Langmuir adsorption isotherm is predicated on the idea that there are no interactions between adsorbed molecules (single-layered adsorption) and that the active sites are uniformly distributed across the adsorbent’s surface. For non-ideal adsorption or multi-layer adsorption, involving systems with heterogeneous surface energy, the Freundlich isotherm is a match in its assumptions. Figure 11A,B show the linearizing plots for Langmuir and Freundlich isotherms, respectively. The raw outputs are also summarized in Table 7. Considering the R^2^ of these two alternative model fits, MB exhibited a higher R^2^ for the Langmuir isotherm, whereas the converse was found for AY that was better fit by the Freundlich isotherm. It can be inferred that single-layer adsorption occurred for MB which had a high solubility in water, without interactions between adsorbed molecules, and the adsorption capacity changed with the initial concentration, while the active sites were uniformly distributed across the adsorbent surface. Unlike this, the adsorption of AY from solution involved systems with heterogeneous surface energy.

In addition, both the Langmuir and Freundlich equations enable the prediction of the maximum adsorption capacity (*q_m_*). As for MB, the *q_m_* estimate obtained from the Freundlich equation was 0.98 mg/g, which did not correlate well with the experimental data (maximum *q_e_* at 200 mg/L of MB was 3.03 mg/g). However, the *q_m_* estimate obtained from the Langmuir equation was 3.15 mg/g, which was approximately that observed. The *q_m_* for MB was more or less the same with *q_e_*, because the solubility of MB in water was less at higher concentrations. On the other hand, the *q_m_* was found differently for AY. The *q_m_* estimate obtained from the Langmuir equation was 2.38 mg/g, which also did not match the experimental data well, as it was clearly less than the amount of AY adsorbed in the experiment (maximum *q_e_* at 200 mg/L of AY was 3.95 mg/g). However, the *q_m_* estimate from the Langmuir equation (10.31 mg/g) was relevant, as the natural rubber latex foam adsorbed more AY when the concentration was >200 mg/L. As can be seen from Figure 6, the removal efficiency remained constant still at a 200 mg/L AY concentration, indicating that natural rubber latex foam can adsorb more even at concentrations beyond 200 mg/L. This did not happen with MB, whose removal efficiency tended to already decrease at the higher concentrations tested.

Alkan et al. [34] further described the *n* in the Freundlich equation. The *n* indicates three different phenomena; 1/*n* = 1 indicates that there is a barrier between the adsorbent and dye solutions and it is concentration-independent, the adsorption is more towards a Langmuir isotherm when 1/*n* < 1, and the adsorption is a combination between Langmuir and Freundlich isotherms when 1/*n* > 1. Here, it can be concluded that the adsorption follows the Langmuir model for MB and a combination of the Langmuir and Freundlich models for AY. The sequence of adsorption can be described as follows: (1) the dye solution moves through the outer surface of the natural rubber latex foam, (2) the dye solution diffuses into the pores of the natural rubber latex foam, and (3) the dye is adsorbed within the pores of the natural rubber latex foam.

**Figure 12:**
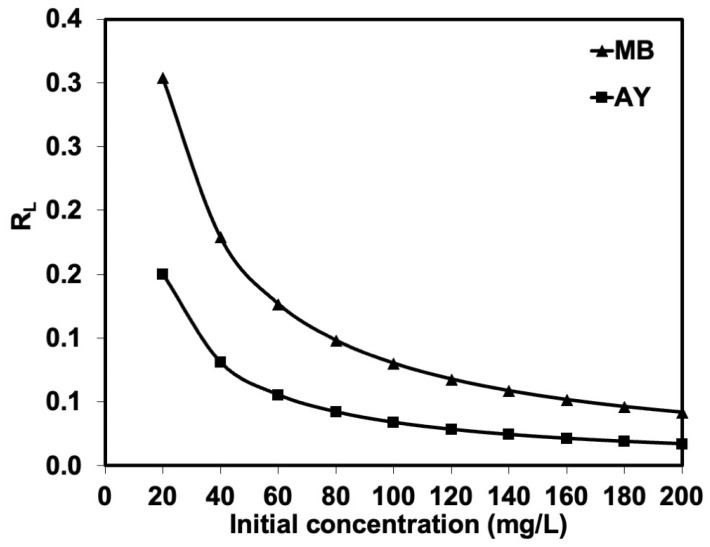
The RL for natural rubber latex foam adsorbing MB or AY.

The adsorption equilibrium constant (*K_L_*) can be further used to calculate the separation factor (*R_L_*) at any concentration (see Figure 12). The *R_L_*, defined as *R_L_* = 1/(1 + *K_L_C*_0_), tended to decrease with the initial MB or AY concentration. It was below 1 (<1), which reflects a favorable adsorption process [35], indicating that the adsorption was very favorable and the adsorbent exhibited good potential. This is where the AY is more favorable to natural rubber latex foam than MB. This is in a good agreement with the previous results, showing that natural rubber latex foam adsorbed more AY than MB from aqueous solutions.

### 3.5. Antibacterial Performance of the Natural Rubber Latex Foam

**Figure 13:**
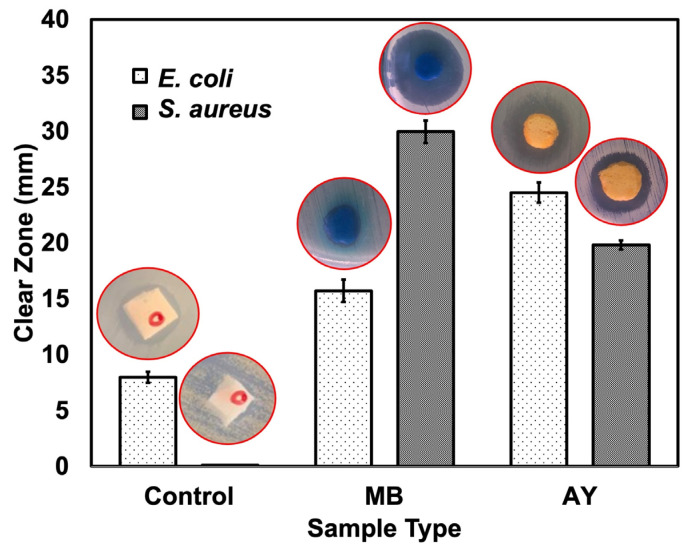
The inhibition zones of the control, and MB- and AY-carrying foam samples against *S. aureus* and *E. coli*.

One of the objectives of this study was to reutilize the dye adsorbent as an antibacterial foam. It was assumed that MB and AY solutions affect living organisms. Therefore, the dye can be used as an antibacterial agent. In this research, antibacterial efficacies were tested qualitatively and quantitatively. The qualitative test measures the radius of bacterial inhibition, while the quantitative assay quantifies the survivors after the sample is mixed with the bacteria at a certain qt. The bacteria used in the test were selected from Gram-negative (*S. aureus*) and Gram-positive (*E. coli*) representatives. The inhibition zones and corresponding images of MB- and AY-carrying foam are shown in Figure 13. It was found that the clean foam (control) did not inhibit *S. aureus*, but it appeared to inhibit *E. coli*. Gram-positive (*S. aureus*) and Gram-negative bacteria (*E. coli*) have different cell wall layers. *S. aureus* contains a peptidoglycan layer thicker than that in *E. coli* [36]. The thicker the cell wall is, the stronger it is. Hence, *S. aureus* inhibition was observed. Moreover, it was also found that a radius of inhibition occurred with the control sample. This is simply due to the presence of ZnO in the rubber foam as an activator. ZnO is known to have antibacterial properties due to its photocatalytic activity [37]. ZnO can generate reactive oxygen species that can destroy the cell walls of bacteria. When considering the antibacterial activity of dye-carrying foam, it was found that it could inhibit both *S. aureus* and *E. coli*. MB has been used as a bacteriostatic agent; it can inhibit the growth of bacteria by interfering with their metabolic processes. It is commonly used in microbiology as a staining agent to visualize bacteria under a microscope. Staining will destroy the cell wall as well. This is in line with research works that used dye-carrying foams as antibacterial agents. Additionally, MB may have some properties that affect bacterial growth indirectly.

**Figure 14:**
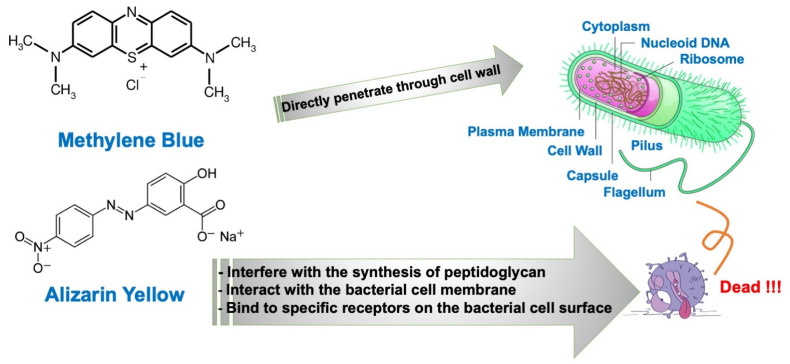
Schematic illustration of the mechanisms by which MB and AY kill bacteria.

The charge of a dye can affect its interactions with bacterial cells, but the killing of bacteria depends on various factors, including the specific mechanism of action of the molecule and the characteristics of the bacterial cell wall [38]. MB is a cationic molecule; it tends to interact more strongly with negatively charged components, such as the cell membranes of *E. coli*. But this did not happen in this case. It is important to note that MB can still interact with *S. aureus*. MB can also penetrate bacterial cells and exert its effects on various intracellular processes, regardless of the bacterial cell type. This can be further explained by the water solubility of MB, as its high water solubility. Water solubility indicates the hydrophilicity of the substance. The outer surface of most bacterial cells is dominated by hydrophilic substances. For instance, the peptidoglycan layer in Gram-positive bacteria is hydrophilic due to the exits of amino acid, while the outer membrane of Gram-negative bacteria contains lipopolysaccharides, which are also hydrophilic [39]. This gives MB the ability to penetrate bacterial cells and destroy them. On the other hand, negatively charged molecules, such as AY or other antimicrobial peptides, can also interact with bacterial cells in different ways. Some molecules with a negative charge can disrupt the integrity of the bacterial cell wall. For example, certain antibiotics like penicillin interfere with the synthesis of peptidoglycan, a component of the bacterial cell wall, leading to cell lysis and death [40]. Negatively charged molecules may also interact with the bacterial cell membrane, causing the permeabilization or disruption of the membrane. This can lead to the leakage of cellular contents and eventual cell death [41,42]. Furthermore, some negatively charged molecules can bind to specific receptors or components on the bacterial cell surface, interfering with essential cellular processes or signaling pathways [43]. The mechanisms of MB and AY in killing bacteria are further illustrated in Figure 14.

The results of the plate count agar test, which is a quantitative antibacterial efficacy test, are shown in Table 8. It was found that the use of dye-carrying foam resulted in a decrease in bacterial survival over time. This demonstrates the ability of the used adsorbent foam to inhibit bacteria. The reduction percentage of bacteria over time is also reported. A positive percentage indicates a higher growth of bacteria. As for the control sample, it was found that the number of bacteria had increased after 24 h. This happened to both *E. coli* and *S. aureus*. The percent survival seemed to be significantly high for *S. aureus*. As mentioned earlier, *S. aureus* has a thicker peptidoglycan layer than *E. coli*. It allows such bacteria to survive better and grow again after a certain period. However, this did not happen with the dye-carrying foam; the percent survival decreased with contact time, and no bacteria of *E. coli* and *S. aureus* survived after 24 h. This is because the MB and AY adhere to the bacterial cell wall and destroy it. From both qualitative (halo test) and quantitative (plate count agar) experiments, it can be concluded that MB- and AY-carrying foams can be utilized as antibacterial foams.

## 4. Conclusions

The natural rubber latex foam was successfully prepared for use as a dye adsorbent with an optimized foam density. MB and AY were used as representative dyes in this study. The foaming volume affected the removal of MB and AY from aqueous solutions. The adsorbed amount was found to be the highest at a 60% foaming volume. However, when the highest foaming volume was used (80%), the adsorbed amount no longer increased. Later, only the natural rubber latex foams prepared to a 60% foaming volume were studied further for batch adsorption, kinetics, and adsorption isotherms. The results clearly show that adsorption increased with initial dye concentration. A kinetic study indicated that the adsorption was well-fitted by pseudo-second-order model. The initial adsorption rate was higher when the concentration of the dye solution was increased. The adsorption had two stages in which MB and AY showed different phenomena, namely, the initial and stabilized stages of adsorption. The adsorption of MB at the former stage was still evolving and did not remain constant, like in the case of AY. This is associated with the water solubilities of MB and AY. The initial stage took a much longer time when a higher initial concentration was tested. The adsorption isotherms of the MB and AY solutions matched both the Langmuir and Freundlich equations. Such an equation enabled estimating the *q_m_* of natural rubber latex foam for MB and AY solutions. The maximum adsorptions of MB and AY from solution were 3.15 and 10.31 mg/g, respectively. In addition, the dye-carrying foam was further utilized as an antibacterial foam. It was clear from both qualitative and quantitative tests that the dye-carrying foams could inhibit bacterial growth of both Gram-positive (*S. aureus*) and Gram-negative (*E. coli*) specimens. It can be concluded that the natural rubber latex foam can serve as a dye adsorbent and be thereafter further utilized as an antibacterial foam.

## Figures and Tables

**Table 1:** The formulation used to prepare natural rubber latex foam.

Ingredient	Amount (phr)
60% HA	100
20% Potassium Oleate	2.0
50% ZDEC	1.0
50% ZMBT	1.0
50% Sulphur	2.5
50% Wingstay™ L	1.5
15% DPG	1.2
50% ZnO	2.0
20% SSF	1.2

**Table 2:** The foaming volumes and percentages while whipping the foam.

Foaming Volume: Beater Volume (ɸ)	Foaming Percentage (%)
0.2:1	20
0.4:1	40
0.6:1	60
0.8:1	80

Remark: 0.1:1 ratio is the original volume of latex compound before whipping into the foam.

**Table 3:** The absorption capacities (*q_e_*) of the natural rubber latex foams prepared to various foaming volumes.

Foaming (%)	*q_e_* (mg/g)	
	MB	AY
20	1.62 ± 0.04	1.78 ± 0.04
40	1.65 ± 0.05	1.85 ± 0.07
60	1.76 ± 0.08	1.97 ± 0.05
80	1.72 ± 0.06	1.93 ± 0.04

**Table 4:** The amount of MB adsorbed, and parameters identified in pseudo-first-order and pseudo-second-order models for natural rubber latex foam adsorbent.

*C_0_* (mg/L)	*q_e_* (exp) (mg/g)	Pseudo-First-Order			Pseudo-Second-Order		
		*q_e_* (mg/g)	*k*_1_ (min^−1^)	R^2^	*q_e_* (mg/g)	*k*_2_ (g/mg·min)	R^2^
20	0.38949	0.02934	0.0026	0.9565	0.389494	0.27044	1
40	0.76245	0.22025	0.0036	0.8276	0.762454	0.055595	0.9997
60	1.10684	0.20633	0.0029	0.9486	1.10684	0.042337	0.9998
80	1.45910	0.42567	0.0034	0.8578	1.4591	0.025327	0.9997
100	1.76027	1.52181	0.005	0.8485	1.76027	0.011993	0.9991
120	2.07299	1.04875	0.0032	0.6595	2.07299	0.009737	0.9977
140	2.18008	0.83535	0.0025	0.8499	2.18008	0.007509	0.9974
160	2.48712	1.55815	0.0032	0.8981	2.48712	0.004452	0.9955
180	2.75528	1.45747	0.002	0.7526	2.75528	0.00298	0.9782
200	3.03301	4.37235	0.0037	0.913	3.03301	0.000551	0.9456

**Table 5:** The amount of AY adsorbed, and parameters identified in pseudo-first-order and pseudo-second-order models for natural rubber latex foam adsorbent.

*C*_0_ (mg/L)	*q_e_* (exp) (mg/g)	Pseudo-First-Order			Pseudo-Second-Order		
		*q_e_* (mg/g)	*k*_1_ (min^−1^)	R^2^	*q_e_* (mg/g)	*k*_2_ (g/mg·min)	R^2^
20	0.3843	0.03344	0.0024	0.845	0.3843	0.00206	0.9998
40	0.7760	0.01417	0.0013	0.9123	0.7760	0.00445	1
60	1.1755	0.01935	0.0028	0.8066	1.1755	0.00454	1
80	1.5712	0.02789	0.0029	0.9472	1.5712	0.00321	1
100	1.9687	0.05013	0.0032	0.9114	1.9687	0.00213	1
120	2.3661	0.04573	0.0027	0.9555	2.3661	0.00193	1
140	2.7654	0.08304	0.0036	0.9757	2.7654	0.00129	1
160	3.1604	0.11894	0.003	0.8517	3.1604	0.00083	1
180	3.5566	0.16372	0.0026	0.7766	3.5566	0.00050	0.9999
200	3.9510	0.13581	0.002	0.9342	3.9510	0.00048	1

**Table 6:** The rate constants of intraparticle diffusion for initial adsorption (*ki*_1_) and for stabilized adsorption (*ki*_2_).

*C*_0_ (mg/L)	*ki* (mg/g·min)^1/2^ of MB		*ki* (mg/g·min)^1/2^ of AY	
	*ki*_1_	*ki*_2_	*ki*_1_	*ki*_2_
20	0.029	0.0007	0.0476	0.0003
40	0.0526	0.0027	0.0977	0.0004
60	0.0784	0.0037	0.1493	0.0007
80	0.1011	0.0058	0.1986	0.0008
100	0.1087	0.0114	0.2496	0.0009
120	0.1115	0.0121	0.2994	0.0011
140	0.1233	0.0146	0.3512	0.0018
160	0.1393	0.0245	0.4000	0.0022
180	0.1414	0.0264	0.4499	0.0028
200	0.1659	0.0594	0.4989	0.0033

**Table 7:** The parameters identified in Langmuir and Freundlich isotherms by fits to natural rubber latex foam adsorbing MB and AY.

Dyes	Langmuir Isotherm Parameters				Freundlich Isotherm Parameters			
	*q_m_* (mg/g)	*K_L_* (L/mg)	*R_L_*	R^2^	*K_F_* (mg/g)/ (mg/L)^1/n^	*q_m_* (mg/g)	1/n	R^2^
MB	3.1486	0.1147	0.0418	0.9751	0.6343	0.98	0.3915	0.9591
AY	2.3832	0.2828	0.0174	0.9398	0.9007	10.31	1.7744	0.9559

**Table 8:** The plate count agar test of the MB- and AY-carrying natural rubber latex foams against *E. coli* and *S. aureus*.

Sample	*E. coli*				*S. aureus*			
	Viable Cell Count (CFU/mL)		Bacteria Survival (%)		Viable Cell Count (CFU/mL)		Bacteria Survival (%)	
	0 h	24 h	0 h	24 h	0 h	24 h	0 h	24 h
Control	2.2 × 10^6^	2.2 × 10^8^	0	+9900	2.2 × 10^6^	3.2 × 10^6^	0	+14,445.4
MB	5.20 × 10^5^	0	0	−100.0	2.00 × 10^6^	0	0	−100.0
AY	1.70 × 10^6^	0	0	−100.0	2.20 × 10^6^	0	0	−100.0

## Data Availability

The data presented in this study are available upon request from the corresponding author.
